# Machine learning and artificial intelligence in cardiac transplantation: A systematic review

**DOI:** 10.1111/aor.14334

**Published:** 2022-06-20

**Authors:** Vinci Naruka, Arian Arjomandi Rad, Hariharan Subbiah Ponniah, Jeevan Francis, Robert Vardanyan, Panagiotis Tasoudis, Dimitrios E. Magouliotis, George L. Lazopoulos, Mohammad Yousuf Salmasi, Thanos Athanasiou

**Affiliations:** ^1^ Department of Cardiothoracic Surgery Imperial College NHS Trust, Hammersmith Hospital London UK; ^2^ Department of Surgery and Cancer, Faculty of Medicine Imperial College London London UK; ^3^ Faculty of Medicine University of Edinburgh Edinburgh UK; ^4^ Department of Cardiothoracic Surgery University Hospital Thessaly Larissa Greece; ^5^ Department of Cardiac Surgery University Hospital of Heraklion Crete Greece

**Keywords:** artificial intelligence, cardiac transplantation, heart transplantation, machine learning

## Abstract

**Background:**

This review aims to systematically evaluate the currently available evidence investigating the use of artificial intelligence (AI) and machine learning (ML) in the field of cardiac transplantation. Furthermore, based on the challenges identified we aim to provide a series of recommendations and a knowledge base for future research in the field of ML and heart transplantation.

**Methods:**

A systematic database search was conducted of original articles that explored the use of ML and/or AI in heart transplantation in EMBASE, MEDLINE, Cochrane database, and Google Scholar, from inception to November 2021.

**Results:**

Our search yielded 237 articles, of which 13 studies were included in this review, featuring 463 850 patients. Three main areas of application were identified: (1) ML for predictive modeling of heart transplantation mortality outcomes; (2) ML in graft failure outcomes; (3) ML to aid imaging in heart transplantation. The results of the included studies suggest that AI and ML are more accurate in predicting graft failure and mortality than traditional scoring systems and conventional regression analysis. Major predictors of graft failure and mortality identified in ML models were: length of hospital stay, immunosuppressive regimen, recipient's age, congenital heart disease, and organ ischemia time. Other potential benefits include analyzing initial lab investigations and imaging, assisting a patient with medication adherence, and creating positive behavioral changes to minimize further cardiovascular risk.

**Conclusion:**

ML demonstrated promising applications for improving heart transplantation outcomes and patient‐centered care, nevertheless, there remain important limitations relating to implementing AI into everyday surgical practices.

## Graphical abstract

Artificial intelligence and machine learning could aid a patient's journey within heart transplantation by predicting the potential benefits of transplantation by analyzing initial lab investigations and imaging, ascertaining graft failure and mortality after transplantation, assisting a patient with medication adherence, and creating positive behavioral changes to minimize further cardiovascular risk.

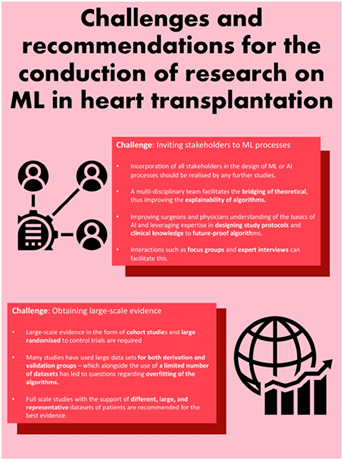

## 1 BACKGROUND

Heart transplantation remains the definitive treatment for patients with end‐stage heart failure. While the number of heart transplants across the world has increased, the supply of heart donors is yet to increase enough to meet the demand; therefore, bringing the issues of resource allocation into question.^1^ The process of graft allocation is complicated, having to consider both patient and donor characteristics in pre‐, peri‐ and post‐operative settings, thus illustrating the multidimensional nature of the matching process. Previous studies in heart transplantation have demonstrated the use of points‐based scoring systems, using a selection of identified variables, in order to predict the main endpoints of mortality and graft failure, but such studies observed poor predictability.^2^ With the increase in demand for donor hearts, prediction of a successful transplantation becomes absolutely paramount, and predictability could be improved by inputting a more extensive and updated donor and recipient information and the utilization of a more powerful analysis, machine learning.^3^

The use of artificial intelligence (AI) has the potential to revolutionize clinical practice. Machine learning (ML) enables the identification of non‐linear relationships and contributing variables that have conventionally been thought to be of limited use.^4^ Utilizing such variables using a ML model allows clinicians to accurately predict prognosis post‐transplantation, quantify the risk of rejection, and ascertain waitlist mortality for those who may not survive long enough to receive a heart, as already illustrated in kidney and liver transplant recipients.^5^, ^6^ Previous studies by the International Society of Heart and Lung Transplantation (ISHLT) have attempted to investigate mortality rates and ascertain the variables most predictive for patient's post‐transplant by utilizing traditional regression models and multivariable analysis.^7^, ^8^ These models remain underutilized in clinical practice due to their relatively weak and variable predictive powers of outcomes that are multidimensional in nature.

ML models can analyze more variables than traditional models to thereby build new co‐variate relationships and identify variables most influential in a particular process. Traditional statistical models aim to ascertain the probability of an event occurring due to a particular variable. Furthermore, ML models allow for a greater number of associated variables to be studied and then build a model based on parameters that influence the outcome the most. In cardiac transplantation, this could guide clinicians in decision making on the allocation of hearts for transplantation, increase accuracy in predicting graft failure and mortality, and predict those at highest risk for rejection post‐transplantation.

This review aims to systematically evaluate the currently available evidence investigating the use of artificial intelligence and machine learning in the field of cardiac transplantation. Furthermore, based on the challenges identified we aim to provide a series of recommendations and a knowledge base for future research in the field of ML and heart transplantation, ultimately aiding patient‐centered care.

## 2 METHODS

### 2.1 Literature search strategy

A systematic review was conducted in accordance with the Cochrane Collaboration published guidelines and the Preferred Reporting Items for Systematic Reviews and Meta‐Analyses (PRISMA) statement. MEDLINE, EMBASE, PubMed, Cochrane, and Google Scholar were searched for original articles from inception to November 2021 that discussed:

- (P) Patients undergoing cardiac transplantation.
- (I) The use of Artificial Intelligence (AI) or machine learning (ML).
- (C) Current algorithms used to predict outcomes, if available.
- (O) Outcomes including mortality, graft failure, and their predictors.

The search terms used included (heart transplantation OR cardiac transplantation OR heart transplant OR cardiac transplant OR heart allograft OR cardiac allograft OR heart heterograft OR cardiac heterograft OR heart homograft OR cardiac homograft) AND (machine learning OR artificial intelligence OR deep learning OR Decision Trees OR Neural Networks). Further articles were identified through the use of the “related articles” function on MEDLINE and a manual search of the references lists of articles found through the original search. The only limits used were the mentioned time frame and the English language.

### 2.2 Study inclusion and exclusion criteria

All original articles were included reporting the use of machine learning or artificial intelligence in cardiac transplantation. Studies were excluded from the review if: (1) inconsistencies in the data impeded extraction of data and (2) the study was performed in an animal model. Reviews, case reports, preclinical studies, and abstracts from meetings were excluded. By following the aforementioned criteria, two reviewers (H.S.P. and J.F.) independently selected articles for further assessment following title and abstract review. A third independent reviewer (A.A.R.) resolved any disagreements between the two reviewers. Potentially eligible studies were then retrieved for full‐text assessment.

### 2.3 Data extraction and critical appraisal of evidence

All full texts of retrieved articles were read and reviewed by two authors (H.S.P. and J.F.) and a unanimous decision was made regarding the inclusion or exclusion of studies. When there was disagreement, the final decision was made by a third reviewer (A.A.R.) Using a pre‐established protocol, the following data were extracted: first author, study design, machine learning technique(s) used, population number, and main outcomes. A data extraction sheet for this review was developed and pilot‐tested using 3 randomly selected included studies and subsequently was refined accordingly. Data extraction was performed by two review authors (H.S.P. and J.F.). The correctness of the tabulated data was validated by a third author (A.A.R).

### 2.4 Risk of bias

**FIGURE 1:**
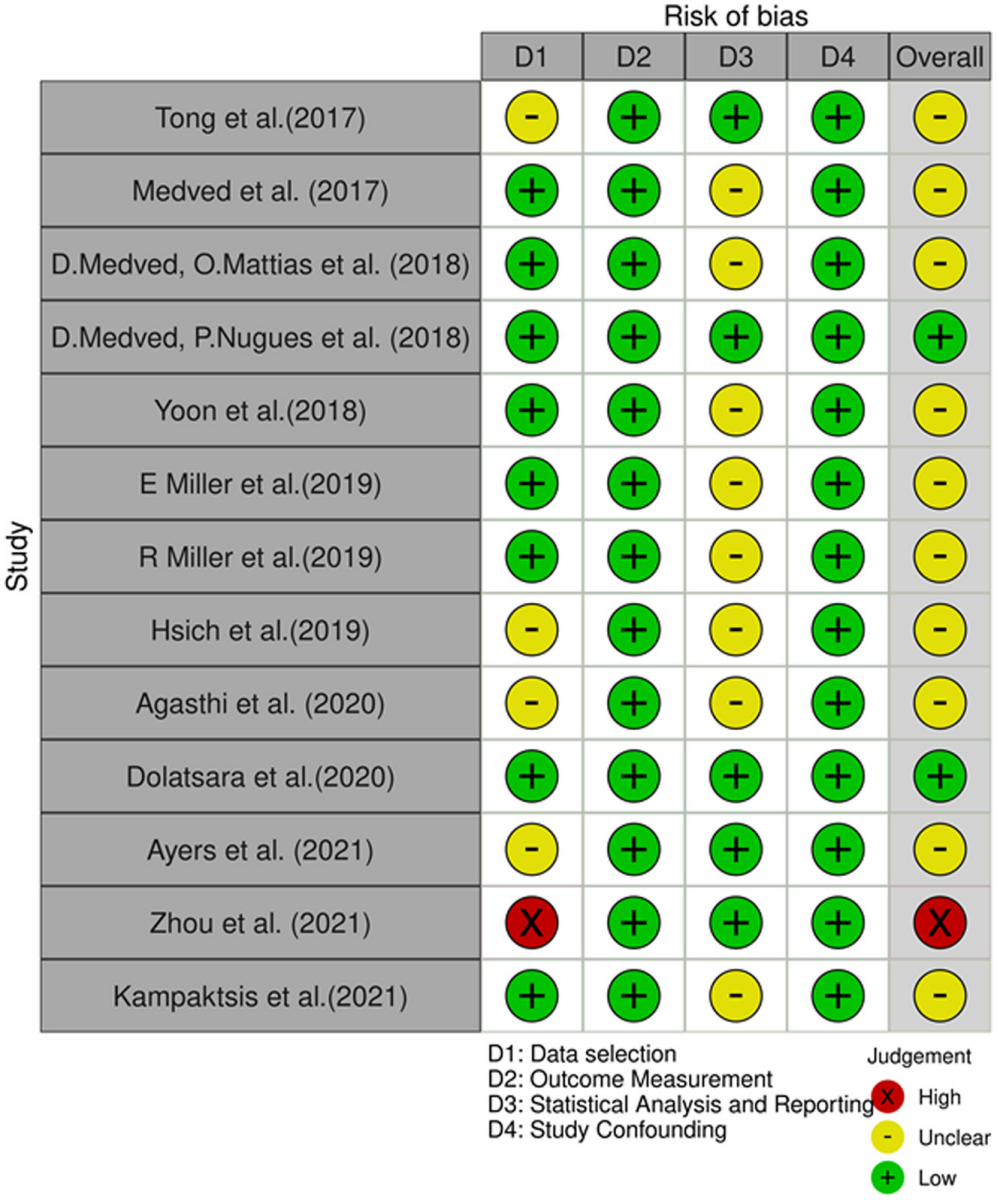
Risk of bias diagram

The risk of bias in the selected articles was evaluated by two independent reviewers (A.A.R. and H.S.P.) using an adapted cochrane collaboration risk of bias tool (Figure 1). The methodological quality of the studies was assessed based of domains: (1) Study Participation, (2) Study Response, (3) Outcome Measurement, (4) Statistical Analysis and Reporting, (5) Study Confounding. An overall grading of low, medium, or high risk of bias was then allocated.

## 3 RESULTS

### 3.1 Study selection

**FIGURE 2:**
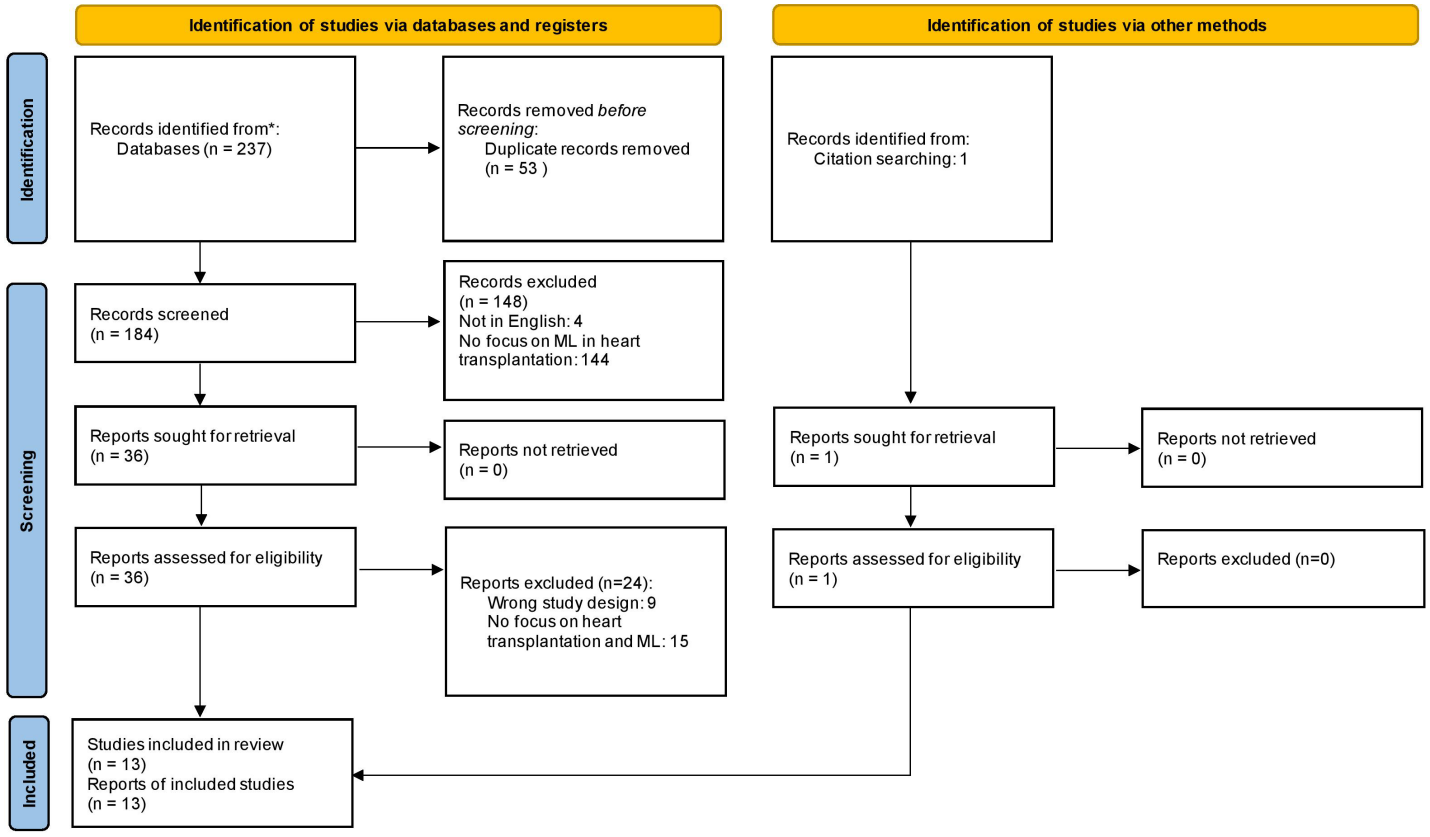
PRISMA flow chart

A total of 237 articles were identified in the literature search, of which 180 were screened following deduplication and were read in full and assessed in accordance with the inclusion and exclusion criteria. A total of 13 studies were included in this review following critical appraisal, featuring 463 850 patients. The entire study selection process is illustrated in Figure 2. A summary of the studies collected and their respective designs, type of outcomes measured, and its implementation as well as the main reported outcomes are found in Table 1.

**TABLE 1:** Summary of the studies included in the systematic review

Study	Type of study; Country; Database used	Algorithm/model/method of implementation	Population number	Aim	Main reported outcomes
Tong et al.^21^	M, P; USA; Whole slides from the Children's Healthcare of Atlanta	Deep Neural Network	43	Automate prediction of heart transplant rejection using histopathological whole‐slide imaging	Shape and distribution of nuclei in tissue images dominate algorithm prediction. NN can significantly reduce overfitting and achieve more stable accuracy compared to NN without regularization and drop out
Medved et al.^11^	M, NP; USA; UNOS	ANN	27 444	Predict outcome 180, 365, 730 days after entering HTx list (Outcome include waiting, transplanted, or dead)	Extracted top 10 variables (weighted by importance) that affect outcome
Medved et al.^10^	M, NP; USA; UNOS	NN (IHTSA and LuDeLTA)	49 566	ML predicts the status of the patient in the que and then the post‐transplant survival	The predicted mean survival for allocating according to wait time was about 4300 days, clinical rules 4300 days, and using neural networks 4700 days
Medved et al.^13^	M, NP; USA; UNOS	IHTSA + IMPACT	27 705	Compare two risk models (IHTSA and IMPACT) to predict short‐ and long‐term mortality after heart transplantation	IHTSA model had improved performance and accuracy compared to the IMPACT model. IHTSA shows better discrimination on one‐year mortality. IHTSA predicts short‐term mortality with greater accuracy than traditional risk‐based models based on logistic regression. AUROC: IHTSA (0.643) and IMPACT (0.608). Calibrated IHTSA (0.688) and IMPACT (0.606)
Yoon et al.^16^	M, NP; USA; UNOS	Trees of Predictors	95 275	Construct a tree of predictors (ToPs) and utilize its predictive power for predicting 3 months, 1‐, 3‐, 10‐year mortality after HTx	AUC for 3‐month was 0.660, while the best clinical risk scoring method only achieved 0.587. ToPs achieved better prediction of both survival and mortality. ToPs identifies the most relevant features and is adaptable to changes in clinical practice
Miller et al.^14^	M, NP; USA; UNOS	ANN, CART, RF	2802	Predict 1‐, 3‐, 5‐ year mortality after pediatric heart transplantation	Good predictive value for mortality but poor sensitivity. Due to lack of registry data, MLs ability to predict mortality post‐transplant is fundamentally limited. AUROC: Calibrated—RF Testing 1 Year (1.25), ANN Testing 1 Year (0.73), CART Testing 1 Year (0.46). RF Testing 3 Years (0.60), ANN Testing 3 Years (0.26), CART Testing 3 Years (0.38), RF Testing 5 Years (0.86), ANN Testing 5 Years (0.20), CART Testing 5 Years (0.33)
Miller et al.^9^	M, NP; USA; UNOS	LR, SVM, RF, Decision Tree, NN	56 477	Develop a risk‐prediction model for assessing 1‐year mortality post‐heart transplantation using ML	Major univariate predictors of 1‐year mortality were consistent with previous findings and included age, renal function, body mass index, liver function tests, and hemodynamics. Machine Learning models showed similarly modest discrimination capabilities compared with traditional models (C‐statistic 0.66, all). The neural network model showed the highest C‐statistic (0.66) but was only slightly superior to the simple logistic regression, ridge regression, and regression with LASSO models (C‐statistic = 0.65, all)
Hsich et al.^19^	M, NP; USA; Scientific Registry of Transplant Recipients (SRTR)	RSF	33 069	Identify variables of importance for waitlist mortality using Random Survival Forests	Strong and weak predictive variables were identified. Complex interactions were identified such as an additive risk in mortality. Most predictive variables for waitlist mortality are in the current tiered allocation system except for eGFR and serum albumin which have an additive risk and complex interactions
Agasthi et al.^17^	M, NP; USA; ISHLT	GBM	15 236	Predict mortality and graft failure 5 years after orthotopic heart transplantation	Model utilized 87 variables in a non‐linear fashion to accurately predict mortality/graft failure. Provided top 10 most influential variables for predicting 5‐year mortality/graft failure. AUROC: Mortality (0.717) and Graft failure (0.716)
Dolatsara et al.^20^	M, NP; USA; UNOS	LR, XGB, LDA, RF, ANN, CART	103 570	First stage—use independent machine learning models to predict transplantation outcomes for each time period. Second stage—Calibrate survival probabilities over time using isotonic regression	First stage produces AUROC between 0.60 and 0.71 for years 1–10. Second stage of calculating survival probabilities guarantees monotonicity
Ayers et al.^12^	M, NP; USA; UNOS	Deep Neural Network, LR, AdaBoost, RF	33 657	Predict 1‐year mortality post orthotopic heart transplantation	Ensemble ML model outperformed traditional risk models in predicting mortality. Model was made from preoperative variables. AUROC: LR (0.649), RF (0.691), DNN (0.691), Adaboost (0.653). Final Ensemble ML Model (0.764)
Zhou et al.^15^	NM, NP; China; Database from Hospital	LR, SVM, RF, XGB, AdaBoost, GBM, ANN	381	Develop a risk‐prediction model for assessing 1‐year mortality post orthotopic heart transplantation using ML	RF model performed optimal predictive power. Top 5 most important variables for short‐term prognosis was ALB, age, LA, RBC, HB level. AUROC: RF (0.801), AdaBoost (0.641), LR (0.688), SVM (0.714), XGBoost (0.769), GBM (0.786), ANN (0.755), Naïve (0.500)
Kampaktsis et al.^18^	M, NP; USA; UNOS	Adaboost, SVM, Decision Tree, KNN, LR	18 625	Develop a risk‐prediction model for assessing 1‐year mortality post orthotopic heart transplantation using ML	Adaboost achieved highest predictive performance. Overall, ML showed good predictive accuracy of mortality after HTx. AUROC: 1 Year—Adaboost (0.689), LR (0.642), DT (0.649), SVM (0.637), K‐nearest neighbor models (0.526). IMPACT (0.569). 1 Year Adaboost (0.689), 3 year Adaboost (0.60528), 5 Year Adaboost (0.6283)

Abbreviations: AdaBoost, adaptative boosting; ANN, artificial neural network; CART, classification and regression tree; GBM, gradient boost machines; HTx, heart transplantation; IHTSA, international heart transplant survival algorithm; KNN, k‐nearest neighbor; LDA, linear discriminant analysis; LR, logistic regression; LuDeLTA, Lund deep learning transplant algorithm; M, multicenter, NM, non‐multicenter, NP, non‐prospective; P, prospective; RF, random forest; RSF, random survival forest; SVM, support vector machine.

### 3.2 Prediction of graft failure and mortality

There were 12 studies that discussed the use of machine learning in predicting mortality in heart transplantation patients,^9^, ^10^, ^11^, ^12^, ^13^, ^14^, ^15^, ^16^, ^17^, ^18^, ^19^, ^20^ comprising 463 807 patients and included a conglomerate of different modeling methods. There was 1 study that discussed the use of machine learning in predicting graft failure in heart transplantation,^17^ the study comprised 15 236 patients.

The outcome of the included studies suggests that AI and ML are generally more accurate in predicting graft failure and mortality than conventional regression analysis. The study by Kampaktsis and colleagues found that ML models generally had good predictive power when assessing 1‐year outcome, but its predictive power declined for later outcomes.^18^ A patient’s journey post‐transplant is complex and most likely to be affected by a variety of multi‐system pathologies observed in the aging population. ML models can only make predictions based on what data is available. As such, more data are required to assess the factors which cause and can predict long‐term outcomes in the post‐heart transplant patient. It was interesting to note that the time horizon played a part in which variables were most predictive, meaning that predictive variables were found to differ for 1‐year mortality compared to 5‐year mortality.^16^ This calls for a wider array of data sets to be collected to accurately model factors that are most influential for specific outcomes, for instance, waitlist mortality versus 3‐year mortality. Despite this, even the current ability of the MI models to predict graft failure and morality is a welcome improvement to the donor graft and recipient matching process and thus provides a more efficient use of the current limited resources and thus reduces waiting times and improving prognosis for patients.

### 3.3 Imaging

There was 1 study that discussed the use of machine learning within an image‐based context in heart transplantation,^21^ this comprised 43 patients. Tong et al. developed a deep neural network that can identify histological slides that fit into rejection and non‐rejection cohorts.^21^ The results yielded far more accuracy than manually determining which slide was to be potentially rejected.

## 4 DISCUSSION

This systematic review explored the data on utilizing AI in a heart transplant setting. Thirteen papers were included in this study to investigate its use in heart transplantation. The majority of papers discussed the use of ML models in accurately predicting mortality and survival post‐transplantation. Others discussed models which predict the risk of rejection pre‐transplant and ML use for predicting waitlist mortality.

### 4.1 ML predictors of graft failure and mortality

#### 4.1.1 Length of hospital stay

Remarkably, ML models have been found to depend more on factors that are not of high importance in traditional statistical models.^22^ Indeed, when predicting graft failure and mortality, donor variables such as age were generally found to be of less importance in ML models, while the length of hospital stay was of high importance.^14^, ^15^, ^17^ In this setting, variables that affect length of stay should be optimized to ensure graft patency and survival, and more data points are required to ensure accurate prognostic predictions. The literature provides no clear explanation for the strong predictive power of length of hospital stay on graft patency and mortality. However, increased complications such as bleeding or incidence of infection, and the severity of such complications are known to lead to an increase in the length of hospital stay.^23^, ^24^ Additionally, the length of hospital stay is difficult to ascertain accurately pre‐operatively. Therefore, further studies are needed to establish the potential predictive factors of these clinical outcomes, and its subsequent predictive potential in mortality and graft failure. However, while the exact relationship between the length of hospital stay and graft mortality could not be explained in the current ML models, there is an unequivocal cost‐benefit of reducing the length of stay for both patients and healthcare systems.^25^

#### 4.1.2 Immunosuppression regimen

Anti‐rejection immunosuppression medications are typically given post‐transplant, but most databases do not collect data on patients' immunosuppression regime.^13^, ^17^ Studies highlighted that this factor was influential in predicting graft failure, more so than predicting mortality. This may be due to such regimes decreasing the chances of host rejection, but causing toxic side effects to the kidney, for example.^26^ The overall toxicity increases mortality but does not affect graft failure as much. It is important to note that many databases did not collect data on patients' immunosuppression regime, perhaps due to the perceived lack of importance, and as such, ML models may pave for more broader data collection to increase the models' prognostic accuracy. Incorporation of patient's immunosuppression regimes will not only aid prognostic accuracy but could also potentially aid with the optimization of immunosuppression regimes for each patient. Episodes of graft rejection are associated with subtherapeutic immunosuppressive drug levels and given the various pharmacodynamic and pharmacokinetic factors that are usually involved, the use of ML algorithms on large datasets would enable a multi‐dimensional analysis of these factors and thus could potentially identify the ideal regime for each patient.^27^

#### 4.1.3 Recipient age and congenital heart disease

Recipient age was also found to be an important predictor as opposed to traditional models, which placed importance on the donor age.^14^, ^15^, ^17^ It is interesting to note that ML models generally had a better predictive power for patients above the age of 60. Most of the population in the databases were over 60, and, therefore, the models built on these data were more suited to patients over that age threshold. Younger patients may have unmeasured variables, including variables influenced by congenital heart disease (CHD), for example, which were unaccounted for in most models. One study highlights that the diagnosis of CHD was the most crucial factor in 1‐, 3‐, and 5‐year mortality.^14^ With the rise in adult CHD prevalence and surgery, and its potential implications in transplantation, more data and models are required to ascertain the utility of AI within specific age groups.^28^ This includes incorporating CHD‐specific variables that would otherwise not be needed in the general adult cardiac patient.

#### 4.1.4 Other major factors

As reported in previous studies, prolonged ischaemic time was also found to be significant in predicting graft failure and mortality.^12^, ^16^, ^17^, ^18^, ^28^ Its influence on 5‐year mortality was not so significant. This is unsurprising given the fact that hearts undergoing prolonged ischemic time were more likely to fail during the initial stages after transplantation; hence, having a higher predictive power for 1‐year mortality than 5‐year mortality. Additionally, donor BMI and recipient BMI were found to increase the risk of graft failure mortality.^11^, ^14^, ^15^, ^17^, ^18^ This is consistent with previously published studies.^29^

Two biomarkers found to have a major influence on graft failure were pre‐transplant creatinine and bilirubin.^12^, ^14^, ^15^, ^17^, ^18^ Previous studies have highlighted this relationship as creatinine and bilirubin are useful indicators to assess overall kidney and liver health, both of which are crucial in cardiovascular health in the post‐transplant patient.^30^ Additionally, transplantation itself could affect kidney function due to reduced renal blood flow and the side effects of potent immunosuppressive drugs post‐transplantation.^31^ Serum creatinine also stands as a biomarker for end‐organ failure, and as such, more identification of biomarkers could pave for predicting the likelihood of organ failure and graft failure by identifying the factors which are conducive to an increase in serum creatinine.

### 4.2 Multi‐level functioning of AI in heart transplant: From the laboratory to post‐transplantation patient care

The use of ML algorithms is not restricted to predicting mortality and graft failure. In combination with other applications, AI and ML could aid a patient's journey within heart transplantation by predicting the potential benefits of transplantation by analyzing initial lab investigations and imaging, ascertaining graft failure and mortality after transplantation, and assisting a patient with medication adherence and creating positive behavioral changes to minimize further cardiovascular risk.

Endomyocardial biopsy is a gold standard investigation to screen for the risk of heart rejection. Due to the time‐consuming nature of screening all histological slides manually, utilizing AI and ML could offer an alternative approach to identifying those at risk of rejection.^32^

Medved et al. discuss the use of AI in the allocation of hearts and predicting waitlist mortality.^10^ Two models were created, one which simulated the removal of a patient from the waitlist and the other to predict survival post‐transplant. The survival of the patients allocated by ML models was also evaluated. The results showed that patients allocated by deep neural networks had reduced waitlist mortality and longer survival post‐transplant.

AI has the potential of assisting patients and clinicians in assessing patient‐specific responses to post‐transplant medication. Previous studies in other fields have investigated the use of ML models simulating patient‐specific responses to treatment, to subsequently indicate what treatments patients should and should not receive. A study by Labovitz et al. investigated the use of an AI application on smartphones to improve patient adherence to anticoagulation.^33^ The utilization of AI to positively impact patient behavior on their adherence to medication management will be of particular use in the post‐transplant patient, due to the myriad of treatments patients receive for immunosuppression, anticoagulation, antihypertension, and others.

### 4.3 Challenges with the implementation of ML

**FIGURE 3:**
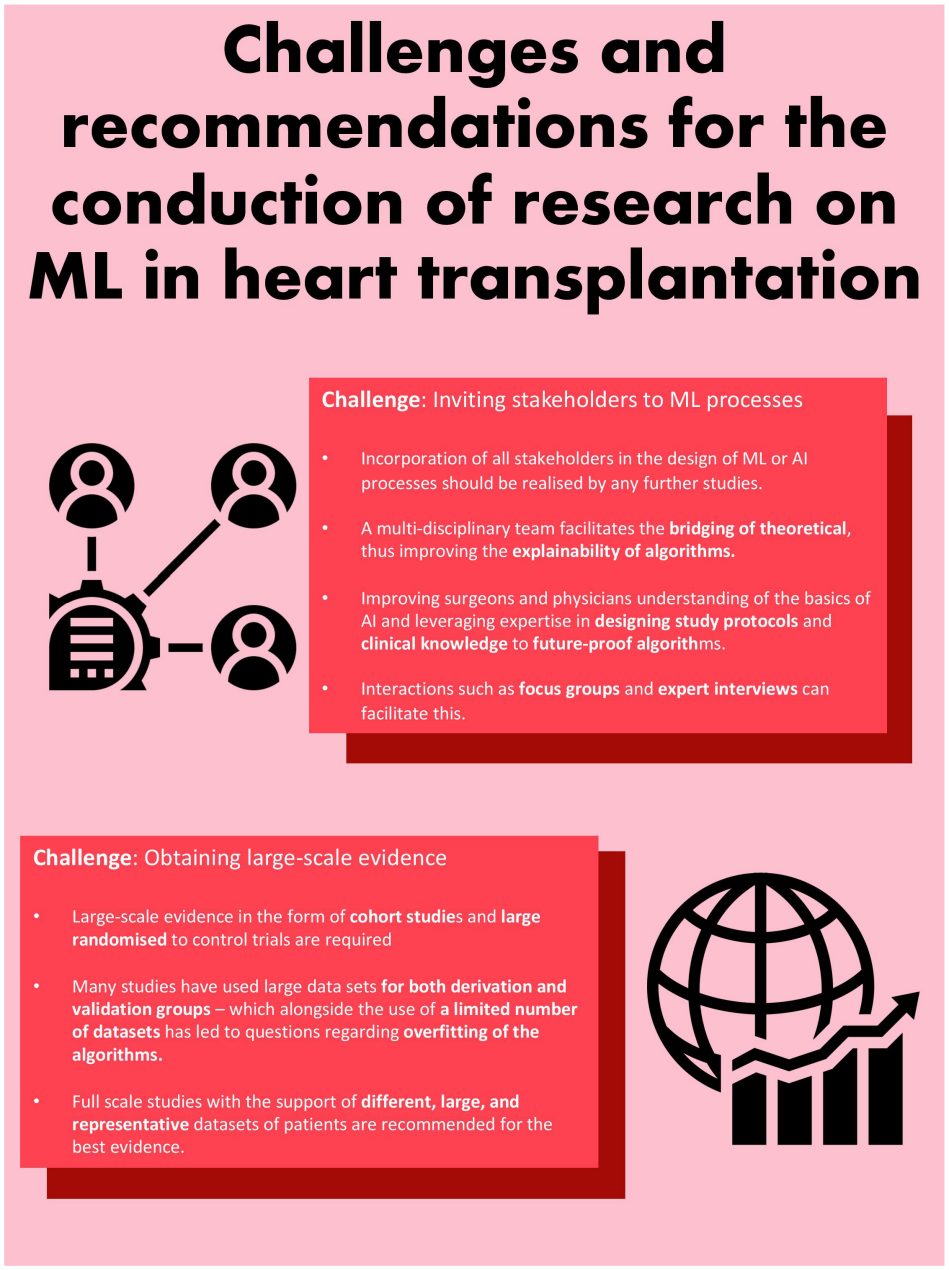
Challenges and recommendations of ML in heart transplantation research.

Despite ML proving to be better performing at predicting endpoints such as mortality and graft failure, both multidimensional in nature, as compared to more traditional methods of scoring systems and regression models, there are a few hurdles currently preventing wider implementation. One such challenge is ascertaining the risk factors for post‐op variables that are identified as predictors of the endpoints (Figure 3). Agasthi et al. discuss the most important factor in mortality and graft failure to be the length of hospital stay—a post‐op variable that is hard to predict prior to transplantation.^17^ Hence, while ML identifies this factor as highly prognostic of the endpoints in question, information regarding the risk factors associated with the length of hospital stay needs to be identified, optimized, and then incorporated into the algorithm. Additionally, the notion of length of the hospital being the strongest predictor of mortality as opposed to donor age also questions the degree of heterogeneity between the models and more importantly, the databases on which they were developed, as well as the methodology that was used, with some studies excluding post‐operative variables.

It must also be noted that the predictive ability of the ML models is as only strong as the initial data set was developed and validated with.^34^ Studies to date have used a range of databases and variation was observed even within the derivation and validation subgroups derived from the same database. Furthermore, what was even more hindering was the number of variables that were removed purely due to a lack of completeness and granularity. A potential reason for this is the fact that many of the databases incorporated data from the 80s and 90s—since then, computational methods, the use of electronic health records, and improvements to data collection guidelines have greatly improved the quality of the datasets with regards to both granularity and validity of the data. Despite this, it is worth noting that the use of registries can still blunt the phenotyping of complex patients which could ultimately affect the predictive ability of ML methods.^9^ Further inspection of the datasets used raises concerns about the homogeneity of the databases—while subgroup analysis was attempted in many of the studies, the nature of data sets, predominantly based on a Caucasian cohort, limits the generalisability of the models. Zhou et al. did demonstrate the effectiveness of ML models for assessing the prognosis of heart transplantation patients in a predominantly Chinese population, however, any consequential contribution to generalisability was limited by the small sample size (381 patients) as well as the focus on the short‐term prognosis, thus highlighting the need for further studies before wider implementation of ML models.^15^

Moreover, the heterogeneity in the ML methods used as well as what the endpoints measured limits comparison between the current studies, and hence the question of what the best model(s) are remains to be solved. The need for constant updatability to the various novel interventions is another area that must be considered when implementing ML algorithms—previous studies have incorporated data from a 30‐ or 40‐year period and in this time, novel interventions such as the left ventricular assist devices have significantly improved patient survival as well as changes to organ allocation sequences.^9^, ^16^ Explainability of ML models also raises reservations with regards to wider implementation—albeit ML models have shown to be highly predictive, this often comes at the expense of explainability to both patients and clinicians and at times, there is a great theoretical and practical divide in the factors identified in machine learning algorithms and the current clinical practice. An example of such mismatch was seen by Zhou et al. who observed smoking to be a protective factor—contrary to previous literature and scientific understanding.^15^

### 4.4 Future steps of AI‐assisted heart transplantation

AI and ML techniques have been proven to generally improve the accuracy of predicting prognosis post‐heart transplantation. Although highly predictive, such models still require a validated dataset. This includes the need for prospective multi‐center studies collecting data on the various elements of heart transplantation. Conventionally, factors such as immunosuppression regime or the causative factors for length of hospital stay were not studied. Our analysis has shown that such factors may pave way for a more powerful predictive capacity for ML models. Such coherent models may allow surgeons of the future to make better decisions on the allocation of hearts, management of patients post‐transplantation, and guide patient in the decision for heart transplantation.

Yoon et al. discuss the use of Tree of Predictors (ToP).^16^ ToPs are predictive models which set binary rules to identify the strongest dependent variables for predictions.^35^ Each tree consists of branches, nodes, and leaves. With nodes having further sub‐nodes. In this case, patients were split into clusters and sub‐clusters based on their specific patient‐donor compatibility features, and a model was created for each specific cluster. This allows for identifying the most relevant covariant for predictive models and utilizes them to ensure greater accuracy in predicting survival pre‐ and post‐transplantation. Greater optimization and personalization for patient decision making in cardiac transplantation will allow for better allocation of resources in a clinical setting. However, these models utilized patients from retrospective studies, and as such, there remains a risk of overfitting the model to prospective patient cohorts, which may not necessarily translate to greater accuracy in current clinical practice.

Further validation and development of ensemble models may allow for a unique website or software, whereby clinicians can input patient variables to calculate the likely prognosis. It is important to note that the results yielded from our studies are likely to be under‐represented the potential of ML models due to the restricted datasets that were inputted. This demonstrates the potential for further multiple non‐linear ML models to be combined to hold a more predictive power for accurately estimating prognosis post‐transplantation. Additionally, the methodology developed in our studies can be applied in other specialties to form a wider application of ML models.

### 4.5 Nationwide data accessibility

Machine learning algorithms are notably data‐driven and perform optimally in scenarios where training models are developed using larger databases. A common issue with smaller databases is that they contain a disproportionately larger quantity of poor data points, as well as outliers and random errors. As a result, they encourage the principle of overfitting whereby a machine learning algorithm models the data to include these erroneous points and incidentally describes random errors rather than the interplay between variables in a dataset. This forces the outcomes of these algorithms to be far less generalizable. One of the key issues, when AI is applied to heart transplantation, remains the lack of detailed data, be it structured or unstructured. Since the number of transplants performed worldwide is relatively low, even the busiest single hospitals will only have hundreds of cases in their registry. In order to take full advantage of this technology, there remains the need for access to nationwide registries which collect data and variables in a granular fashion.

### 4.6 Limitations

This systematic review comes with certain limitations. Much of the data included in this systematic review were from retrospective observational studies, which is conducive to bias and confounding. Additionally, due to the different databases utilized by each individual study, a meta‐analysis is unachievable due to the heterogeneity in the variables included, and due to the type of ML models that were utilized. To test the full potential of ML models and AI, larger multi‐center prospective studies are needed. Further studies will need to consider a broader range of variables, especially those which are commonly not included due to the perceived lack of importance—for example, the immunosuppression regime post‐transplantation.

Due to the timeliness of the following review and in view of the recent rapid advances in the field, we started the following work aiming to be able to rapidly provide the readers with a high‐quality review of this important topic. Therefore, we did not initially register the protocol of our work on Prospero. Although we had taken steps before commencing this review to scan the literature for any ongoing or existing reviews on this topic, not finding any similar work being present, we understand the importance of registering protocols of systematic reviews on PROSPERO to avoid duplication and overlapping works, and the following remains a limitation of this review.

## 5 CONCLUSION

The implementation of machine learning models in heart transplantation has illustrated the scope for this powerful tool which could greatly enhance current clinical practice by improving the predictability of outcomes. Several studies demonstrated the use of machine learning was superior to traditional models of scoring systems and regression models in predicting endpoints of heart transplantation, thus proving vital to improving the chances of successful transplantation and the chances of a successful donor‐recipient match. The use of ML algorithms is not restricted to predicting mortality and graft failure. In combination with other applications, AI and ML could aid a patient's journey within heart transplantation by predicting the potential benefits of transplantation by analyzing initial lab investigations and imaging, ascertaining graft failure and mortality after transplantation, and assisting a patient with medication adherence and creating positive behavioral changes to minimize further cardiovascular risk. Nevertheless, this study also identified the need for higher quality, more granular, and extensive databases since the models are only as good as the initial information that is fed into them. Crucially, the heterogeneity in data restricted the use of such models to adults over the age of sixty. More multi‐center prospective and nationwide datasets are required to address these concerns whereby parameters involved in heart transplantation are collected, regardless of the traditionally perceived importance.

## AUTHOR CONTRIBUTIONS

Arian Arjomandi Rad, Hariharan Subbiah Ponniah, and Vinci Naruka: Concept/design, data collection, data interpretation, drafting article, critical revision of article, approval of article. Jeevan Francis: data curation, drafting article, approval of article. Robert Vardanyan, Panagiotis Tasoudis, Dimitrios E. Magouliotis, George L. Lazopoulos, Mohammad Yousuf Salmasi, and Thanos Athanasiou: Concept/design, data interpretation, critical revision of article, approval of article.

## CONFLICT OF INTEREST

The authors report no relationships that could be construed as a conflict of interest.

## Data Availability

The data that support the findings of this study are available from the corresponding author, upon reasonable request.
